# Methods of determining titanium dioxide nanoparticles enhance inorganic arsenic bioavailability and methylation in two freshwater algae species

**DOI:** 10.1016/j.mex.2018.06.004

**Published:** 2018-06-12

**Authors:** Zhuanxi Luo, Zhenhong Wang, Yameng Yan, Jinli Li, Changzhou Yan, Baoshan Xing

**Affiliations:** a Key Laboratory of Urban Environment and Health, Institute of Urban Environment, Chinese Academy of Sciences, Xiamen 361021, China; b Stockbridge School of Agriculture, University of Massachusetts, Amherst, MA 01003, USA; c College of Chemistry and Environment, Fujian Province Key Laboratory of Modern Analytical Science and Separation Technology, Minnan Normal University, Zhangzhou 363000, China

**Keywords:** Association of inorganic As and nano-TiO2 in algae, Bioaccumulation, Engineered Nanomaterials, Dissociation, Algae, Trojan horse

## Abstract

We developed the effect of titanium dioxide nanoparticles (nano-TiO_2_) on the bioaccumulation and biotransformation of arsenic (*As*), which remain largely unknown. We thus exposed two freshwater algae (*Microcystis aeruginosa* and *Scenedesmus obliquus*) to inorganic *As* with the aim of increasing our understanding on *As* bioaccumulation and methylation in the presence of nano-TiO_2_. Direct evidence of TEM and EDX image showed that nano-TiO_2_ (anatase) entered the exposed algae. Thus, nano-TiO_2_ as carriers boosted arsenic accumulation and methylation in these two algae species, which varied with both inorganic *As* speciation and algae species. Specifically, nano-TiO_2_ could enhance markedly arsenate accumulation in *M. aerugginosa* and arsenite accumulation in *S. obliquus*. Similarly, we found higher content of *As* methylation in *M. aeruginosa* of arsenite with 2 mg L^−1^ of nano-TiO_2_ treatment and in *S. obliquus* of arsenate treatment. Additionally, *S. obliquus* exhibited higher *As* methylation compared to *M. aeruginosa*, being more sensitive to *As* associated with nano-TiO_2_ than *M. aeruginosa*. Due to changes in pH levels inside these exposed algae, the *As* dissociation from nano-TiO_2_ inside algal cell enhanced *As* methylation. Accordingly, the potential influence of nanoparticles on the bioaccumulation and biotransformation of their co-contaminants deserves more attention.

• Nano-TiO_2_ entry is assumed to promote *As* accumulation into exposed algae.
• Nano-TiO_2_ had different carrying capacities for different forms of *As* and algae.
• *As* dissociation from nano-TiO_2_ is assumed to enhance *As* methylation in algae.

## Graphical abstract

**Figure:**
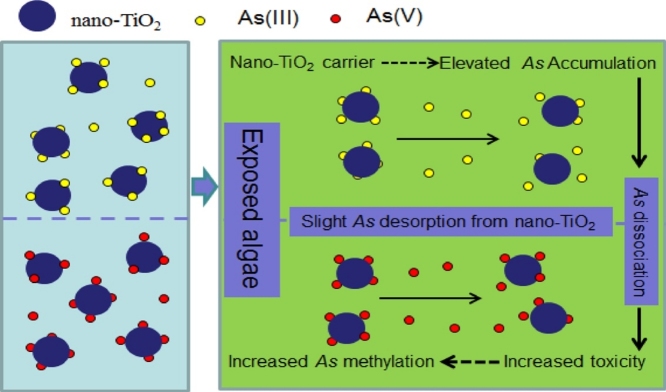

**Table:** 

Subject area	*Select one of the following subject areas:* *Agricultural and Biological Sciences* *Engineering* *Environmental Science*
More specific subject area	*Combination pollution (Nanoparticles)*
Method name	***Association of inorganic As and nano-TiO_2_ in algae***
Name and reference of original method	*If applicable, include full bibliographic details of the main reference(s) describing the original method from which the new method was derived.*
Resource availability	If applicable, include links to resources necessary to reproduce the method (e.g. data, software, hardware, reagent)

## Method

### Nano-TiO_2_ and arsenic preparation

We used the anatase form of nano-TiO_2_ purchased from the Sigma-Aldrich Corporation with a particle size of less than 25 nm and a purity of > 99.7%. A nano-TiO_2_ stock suspension (1 g L^-1^) was prepared by first suspending nanoparticles in ultrapure water. We then sonicated the solution at 33 W for 30 min. The average hydrodynamic size of nano-TiO_2_ was 193 ± 10 nm, as measured by the dynamic light scattering technique (DLS, Malvern Instruments, UK) at automatic attenuator mode. Experimental nano-TiO_2_ concentrations of 100 μg L^−1^ and 2 mg L^−1^ were diluted from the stock suspension. The aggregate morphology of nano-TiO_2_ was observed by a scanning electron microscope (SEM, S-4800, Hitachi, Japan). We used Na_3_AsO_4_·12H_2_O and NaAsO_2_ to prepare *As* stock solutions at 1 mM, which were stored at 4 °C in the dark until further use. Additionally, we measured the average hydrodynamic diameter (d_H_) and zeta potential (ζ) using DLS, and pH levels of nano-TiO_2_ in BG-11 culture media with *As*(III) and *As*(V) of 10 μM at 0.1 mg L^−1^ and 2 mg L^-1^ TiO_2_ concentrations.

### Exposed algae species

The two freshwater alga species (*S. Obliquus* and *M. Aeruginosa*) used in our experiments were inoculated under sterile conditions in BG-11 media in Erlenmeyer flasks at 25 °C. The light-dark cycle used was 16:8 with a light intensity of 115 μmol photons m^2^ s^−1^. For the following experiments, exposed algae were shaken at 100 rpm using a shaker to avoid settling.

### Algae toxicity and stress

Toxicity of *As*(III) and *As*(V) was determined using 96 h growth rate bioassays. Algal cell at the exponential growth phase were added separately into a final concentration of *As*(III) (10 μM) and *As*(V) (10 μM) under increasing nano-TiO_2_ levels (from 0 to 200 mg L^-1^). The initial cell density of exposed algae was 10^6^ cells mL^-1^. We used a hemocytometer and a microscope to measure algal cell density every 24 h until the end of the exposure experiment. The specific growth rate (μ) of cells was thus calculated according to the method reported by Zeng et al. [1]. Afterwards, the 96 h EC50 was calculated based on μ values of tested algal cells using a probability unit graphical method [2].

We examined alga stress from nano-TiO_2_ associated with 10 μM of *As*(III) or *As*(V). Final concentrations of nano-TiO_2_ were 0 mg L^-1^ (control), 0.1 mg L^-1^, and 2 mg L^-1^. Cells at the exponential growth phase were added individually to *As*(V) and *As*(III) under the three separate aforementioned nano-TiO_2_ concentrations. The initial cell density was 1 × 10^6^ cells mL^-1^. At the same time, we conducted parallel experiments with final nano-TiO_2_ concentrations of 0 mg L^-1^, 0.1 mg L^-1^, and 2 mg L^-1^ (without the addition of *As*). We conducted Chl-a quantification after 96 h exposure. Additionally, we measured methane dicarboxylic aldehyde (MDA) to indicate the degree of lipid peroxidation (LPO) in this experiment, representing alga stress from *As* associated with nano-TiO_2_. We detected MDA content using the thiobarbituric acid reactive substances (TBARS) method by applying a reagent kit (the Nanjing Jiancheng Biotechnology Institute, China) according to the manufacturer’s instructions [3].

**Fig. 1:**
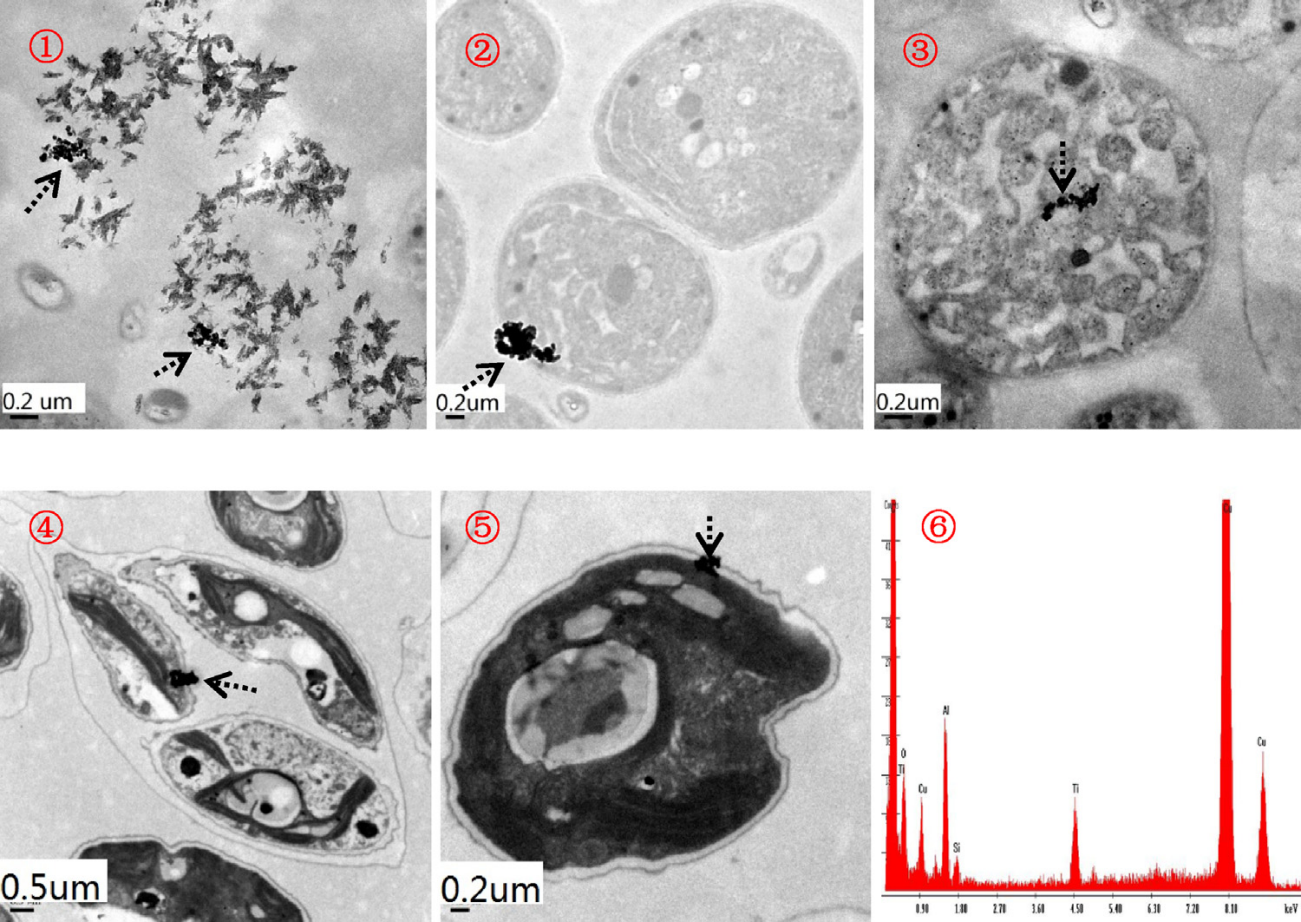
Morphology of titanium dioxide nanoparticles in algae and culture media. Note: Arrows point to nano-TiO_2_.  Nano-TiO_2_ in algae culture media;  and  nano-TiO_2_ in *Microcystis aeruginosa*, indicating that nano-TiO_2_ can enter this algae cell;  and  nano-TiO_2_ in *Scenedesmus obliquus*, indicating that nano-TiO_2_ can enter this algae cell;  EDX analysis of areas pointed by arrows was confirmed to be nano-TiO_2_.

Furthermore, we characterized nano-TiO_2_ in algae and the culture media after both 0.1 and 2 mg/L of nano-TiO_2_ exposure associated with 10 μM of *As*(III) and *As*(V) using TEM as well as energy-dispersive X-ray spectroscopy (TEM, H-7650, Hitachi, Japan; EDX, Genesis XM2) [4]. In brief, we fixed exposed algal cells using 2.5% glutaraldehyde and then refrigerated them for 12 h. Afterwards, the treated algal cells were washed thrice in a 0.1 M phosphate buffer (pH 7.0), then postfixed in 1% osmium tetroxide for 1 h, dehydrated through a graded series of ethanol (30%, 50%, 70%, 90%, 95%, and 100%) and embedded for 12 h. The resultant algal cells were incised using a diamond blade in an ultramicrotome (Leica UC7, Germany) to obtain cell ultrathin sections (approximately 70 nm in thickness) for TEM and EDX observations without staining. Direct evidence of TEM image and EDX observations was provided in Fig. 1.

### Dissociation of inorganic As from nano-TiO_2_ in algae cell homogenates

#### Inorganic arsenic dissociation in algal cell homogenates

**Fig. 2:**
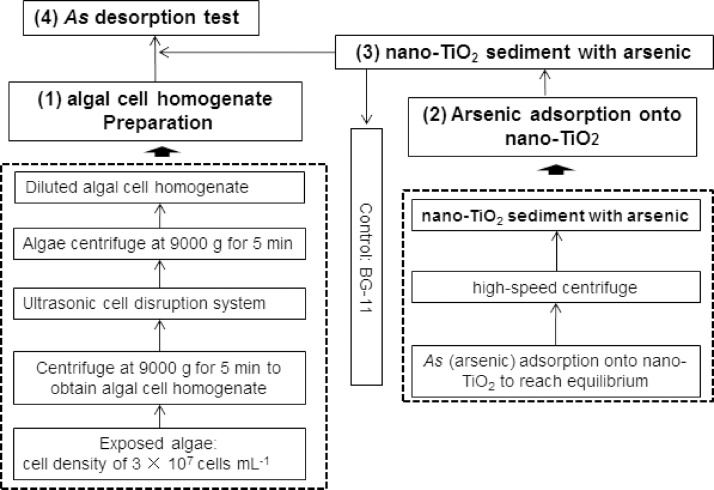
Flowchart of desorption of inorganic *As* from nano-TiO_2_ in algal cell homogenates.

In order to identify whether *As* dissociated from nano-TiO_2_ inside algal cells, we conducted desorption experiments to detect the potential dissociation of inorganic *As* from nano-TiO_2_ in algal cell homogenates, which followed the flowchart provided in Fig. 2. This information could provide insight into oxidative stress of *As* entry into algal cells facilitated by the presence of nano-TiO_2_. In this experiment, the two algal species selected were cultured to a cell density of 3 × 10^7^ cells mL^-1^. We collected an alga pellet in a 50 mL centrifuge tube using a high-speed centrifuge (9000g) for 5 min. BG-11 was then added into the tube containing the alga pellet, and the tube was shaken to re-suspend the species. After 3 min of disruption using an ultrasonic cell disruption system, the algal cell homogenate was separated at a centrifugal force of 9000g for 5 min to remove algae residue, such as cell wall material. Finally, the algal cell homogenate was diluted with BG-11 to 100 mL for further usage. At the same time, we measured algal cell homogenate pH levels and total organic carbon (TOC) to understand the potential mechanisms of the dissociation.

Our preliminary results showed that *As* adsorption on nano-TiO_2_ rapidly reached equilibrium (within 1 h) in the BG-11 medium. In turn, the solution of *As* associated nano-TiO_2_ was prepared 1 h before use to make equilibrated adsorption of *As*(V) and *As*(III) onto nano-TiO_2_. The final concentration of nano-TiO_2_ and *As* was 2 mg L^-1^ and 10 μM, respectively, diluted from their stock solutions with BG-11 for *As* adsorption onto nano-TiO_2_ experiment. After mixing *As* and nano-TiO_2_ for 1 h, the solution was centrifuged for 10 min at 12 000g to obtain the sediment of nano-TiO_2_ associated with *As* by removing the supernatant. We then added 40 mL of the algal cell homogenate and BG-11 (used as the control) into the abovementioned sediment, respectively, to conduct the *As* desorption test in the algal cell homogenate. After shaking for 2 h at 180 rpm min^1^ in a shaker, we obtained the supernatant using a high-speed centrifuge (12 000g). We used 1 mL of the supernatant to measure *As* concentrations using inductively coupled plasma mass spectrometry (ICP-MS 7500a, Agilent). The difference in the final *As* concentration between BG-11 and the algal cell homogenate was the apparent *As* desorbed from nano-TiO_2_, caused by the algal cell homogenate. Three replicates were prepared for this test.

#### Inorganic arsenic depuration from algae

On the other hand, we examined the inorganic *As* depuration from the algae species to further identify whether the dissociation of *As* from nano-TiO_2_ inside algal cells occurred. The two selected alga species, with an initial cell density of 10^6^ cells mL^-1^, were pre-exposed to *As* separately for 96 h in the presence of nano-TiO_2_, from which the final exposure concentrations were 1 μM *As*(V) and 0.8 μM *As*(III) in the presence of 20 mg L^-1^ nano-TiO_2_ for *M. aeruginosa* and 0.1 u M *As*(V) and 0.08 μM *As*(III) in the presence of 2 mg L^-1^ nano-TiO_2_ for *S. obliquus*. Under these exposure concentrations, algae did not exhibit any toxic effects. Moreover, *As*(III) and *As*(V) can absorb onto nano-TiO_2_ by 98.91% ± 0.91% and 98.23% ± 0.87%, respectively. Thus, the *As* accumulated in exposed algae was largely associated with nano-TiO_2_. The As solution was prepared by the same method described above, that is, it was prepared 1 h before use to equilibrate *As*(V) and *As*(III) adsorption on nano-TiO_2_. We then individually centrifuged each pre-exposed algae species into a pellet at 3800g for 10 min. Afterwards, we used sterile ultrapure water (18.2 mΩ cm^−2^) to twice wash each algal pellet before immersing each in an ice-cold phosphate buffer (1 mM K_2_HPO_4_, 5 mM MES, and 0.5 mM Ca (NO_3_)_2_) for 15 min to completely remove apoplastic *As* [5]. The collected pellets were then individually re-suspended in BG-11 media. After 12 h, 5 mL of supernatant was collected at 3800 for 10 min to measure total *As* and titanium (Ti) in media using ICP-MS [6,7]. At the same time, the desorption of *As* from nanoparticles in the absence of algae was comparably performed under the same treated conditions with the exception of using 12 000g centrifugation for 10 min, which further confirmed the depuration of *As* from algal cells. Moreover, we did not find any *As* desorbed from nano-TiO_2_. Thus, the amount of total *As* in the supernatant was the *As* depuration from the algae.

### Inorganic arsenic accumulation and methylation in the exposed algae

An initial cell density of 10^6^ cells mL^-1^ was diluted from cell inoculum during the exponential growth phase. After being inoculated with *M. aeruginosa* or *S. obliques*, each culture was treated immediately and separately with *As*(V) and *As*(III) at 10 μM and the three different concentrations of nano-TiO_2_ (0 mg L^-1^, 0.1 mg L^-1^, and 2 mg L^-1^, respectively) throughout a 4 d uptake period to evaluate the effects of *As* bioaccumulation and methylation affected by the presence of nano-TiO_2_. Cultures devoid of nano-TiO_2_ (0 mg L^-1^) were used as controls. Each *As*(III) treatment was then monitored every 24 h by sampling 5 mL of the aliquot test solution until the end of experiment to detect whether *As*(III) oxidized into *As*(V). Finally, we individually collected two algal solutions of 25 mL each from separate flasks for all treatments, after which we centrifuged them into a pellet at 3800g for 10 min. Afterwards, two 5 mL supernatants were individually collected to measure total *As* and *As* speciation in the media, respectively, to calculate the bioconcentration factor (BCF) and to quantify *As*(III) oxidation in tested suspensions. Furthermore, before being immersed in the aforementioned ice-cold phosphate buffer for 15 min to completely remove apoplastic *As*, both algal pellets were washed twice using sterile ultrapure water (18.2 mΩ/cm^2^) [5]. Afterwards, the two algal pellets were respectively digested after one pellet was dried under warm conditions at 60 °C in an oven while the other was freeze-dried in a vacuum freeze dryer. At this point, total intracellular *As* was measured to determine *As* bioaccumulation using the oven-dried algae samples, while *As* speciation was analyzed to determine *As* methylation using the freeze-dried algae samples, including inorganic *As*, *As*(V), monomethylarsonic acid (MMA), and dimethylarsinic acid (DMA) [8]. The methylated amount of *As* inside algae was calculated as the sum of MMA and DMA in the exposed algae.

### Arsenic determination in algae and medium

We determined total *As* and *As* speciation according to the previous methods used for algal sample preparation and *As* analysis [9]. In brief, approximately 0.02 g of oven-dried algal samples were treated overnight by means of microwave assisted digestion. Afterwards, samples were further diluted to measure *As* using ICP-MS. We had a good recovery rate (92.3% ± 5.6%) using a standard reference sample (GBW08521, the National Research Center for Standard Materials of China). Additionally, approximately 0.02 g of the freeze-dried algal samples was treated overnight. After microwave assisted digestion, samples were filtered using 0.45 μm filters. We then used HPLC-ICP-MS (Agilent LC1100 series coupled with the Agilent ICP-MS 7500a) to measure *As* speciation in algal extracts and media.

### Calculation and statistics

The measured *As* bioaccumulation in algae included free *As* in algal cells and its association with nanoparticles on cell surface and internalized cells. We then used the dry weight of BCF to estimate *As* bioavailability affected by the presence of nano-TiO_2_, which was calculated using the following equation:

BCF = the *As* concentration in algae (μg/g dry weight)/the *As* concentration in media (which includes the *As* adsorbed on nanoparticles; μg L^-1^) × 1000 (1)

Moreover, SPSS 12.0 was used to perform statistical analysis on the data. The data are shown as means with standard deviations (SD). The differences within treated groups were evaluated by two-way analysis of variance (ANOVA) with a least significant difference (LSD) range test at *P*<0.05 significant levels.

## Conclusion

Nano-TiO_2_ promoted the accumulation and methylation of inorganic *As* in the two selected freshwater algae species investigated in this study. Evidence showed that nano-TiO_2_ promotes *As* accumulation into exposed algae species. The dissociation of inorganic *As* from nano-TiO_2_ contributes to its increased toxic effects on exposed algae, which be demonstrated by Chl-a and MDA. Subsequently, stress levels increased as a result of this *As* and nano-TiO_2_ dissociation, thus potentially leading to greater *As* methylation in algae species. It is clear that *As* metabolism is variable between *As* forms and algae species as well as nanoparticles and environmental factors. Furthermore, *As* contamination is globally recognized as being high risk.
